# Systemic inflammation is an important risk factor and predictor of graft loss and mortality one year after kidney transplantation

**DOI:** 10.3389/fimmu.2025.1529812

**Published:** 2025-02-19

**Authors:** Torbjørn F. Heldal, Anders Åsberg, Thor Ueland, Anna V. Reisæter, Søren E. Pischke, Tom E. Mollnes, Pål Aukrust, Finn P. Reinholt, Anders Hartmann, Kristian Heldal, Trond G. Jenssen

**Affiliations:** ^1^ Department of Internal Medicine, Telemark Hospital Trust, Skien, Norway; ^2^ Institute of Clinical Medicine, University of Oslo, Oslo, Norway; ^3^ Department of Transplantation Medicine, Oslo University Hospital – Rikshospitalet, Oslo, Norway; ^4^ Norwegian Renal Registry, Oslo University Hospital – Rikshospitalet, Oslo, Norway; ^5^ Department of Pharmacy, University of Oslo, Oslo, Norway; ^6^ Thrombosis Research Center (TREC), Division of Internal Medicine, University Hospital of North Norway, Tromsø, Norway; ^7^ Research Institute of Internal Medicine, Oslo University Hospital - Rikshospitalet, Oslo, Norway; ^8^ Department of Immunology, University of Oslo and Oslo University Hospital, Oslo, Norway; ^9^ Department of Anesthesiology, Division of Emergencies and Critical Care, Oslo University Hospital, Oslo, Norway; ^10^ Research Laboratory, Nordland Hospital Bodø, Bodø, Norway; ^11^ Section of Clinical Immunology and Infectious Diseases, Oslo University Hospital – Rikshospitalet, Oslo, Norway; ^12^ Department of Pathology, Oslo University Hospital, Rikshospitalet, Oslo, Norway; ^13^ Institute of Health and Society, University of Oslo, Oslo, Norway

**Keywords:** kidney transplantation, graft loss, mortality, inflammation, biomarkers, prediction

## Abstract

**Background:**

An inflammatory environment following kidney transplantation is associated with increased risk of graft loss and mortality, however, evaluation of systemic inflammation is not implemented in structured risk assessment in kidney transplant recipients. Long-term results after transplantation are not satisfactory, and thus tools addressing these issues are needed. In this study, we tested the associations and predictive abilities of a predefined systemic inflammation score one year after transplantation on death-censored graft loss and mortality.

**Methods:**

We included 805 patients who underwent kidney transplantation between 2013 and 2017 at the Oslo University Hospital, Rikshospitalet. The inflammation score included five specifically selected biomarkers known to reflect various inflammatory pathways and to be associated with adverse outcomes following transplantation. The score was assessed in relation to outcomes in models with established risk factors. Discriminatory analyses were performed using Harrell´s C-statistic, and model assessment were evaluated using internal validation, calibration, and likelihood ratio tests.

**Results:**

The median follow-up time was 6.4 years. There were 168 deaths (20.9%) and 42 graft losses (5.2%). The inflammation score one year after transplantation was significantly associated with graft loss (P<0.001) and mortality (P<0.001). The diagnostic performance of the model for graft loss revealed a c-statistic of 0.77 both with and without histological data. The diagnostic performance for mortality displayed a c-statistic of 0.79. In all tested scenarios, the model fit significantly improved after including the inflammation score.

**Conclusions:**

These results suggest a strong association between systemic inflammation one year after transplantation and both graft loss and mortality. Predictive models including the inflammation score and established risk factors were particularly informative when considering mortality. Evaluation of systemic inflammation using this score could be an important tool for risk-assessment after transplantation.

## 1 Introduction

Inflammation is an established risk factor for morbidity and mortality, including cardiovascular diseases and cancer (1–3). Among kidney transplant recipients, inflammation early after transplantation is associated with both kidney allograft loss and mortality (4–7), as well as with the development of post-transplant diabetes mellitus (PTDM) (8). However, these results have not been sufficiently validated, and thus the recipients´ inflammatory profile is yet to be implemented in daily clinical risk assessment.

The improvement in graft survival has decelerated during the last 20 years, and the long-term results are still not satisfactory (9). The iBox Scoring System is the gold standard for predicting the risk of kidney allograft loss and consists of markers reflecting allograft function, the recipients´ immunological response, and histological features (10, 11). The scoring system has shown superior prognostic performances compared with the presence of biopsy-proven acute rejection within the first year after transplantation (12, 13). T-cell mediated rejection (TCMR) and antibody-mediated rejection (AMR) have traditionally been considered the two main classes of transplant rejection, but novel findings including innate allorecognition through macrophages and natural killer cells have highlighted gaps in the current classification and understanding of rejection (14, 15). In contrast to graft survival, there are no established risk scores with an acceptable discrimination ability for predicting mortality among kidney transplant recipients.

Implementation of non-invasive biomarkers into the risk assessment of kidney transplant recipients is desirable, as protocol biopsies are associated with both risks and resource demands. Biomarkers that display predictive abilities regarding both graft loss and mortality are of particular interest. Another important feature is biomarkers that reflect pathways with potential treatment options. We have previously demonstrated associations between systemic inflammation scores ten weeks after transplantation and both long-term graft loss and mortality (4, 5). The objective of this study was to assess whether a specified version of our previously established systemic inflammation score, determined in a new cohort one year after transplantation, added information and enhanced the prediction of both long-term death-censored graft loss and mortality. Moreover, in the present study we investigated patients who underwent transplantation between 2013 and 2017 and, accordingly, were more comparable with today’s clinical practice.

## 2 Materials and methods

### 2.1 Study population and design

**Figure 1:**
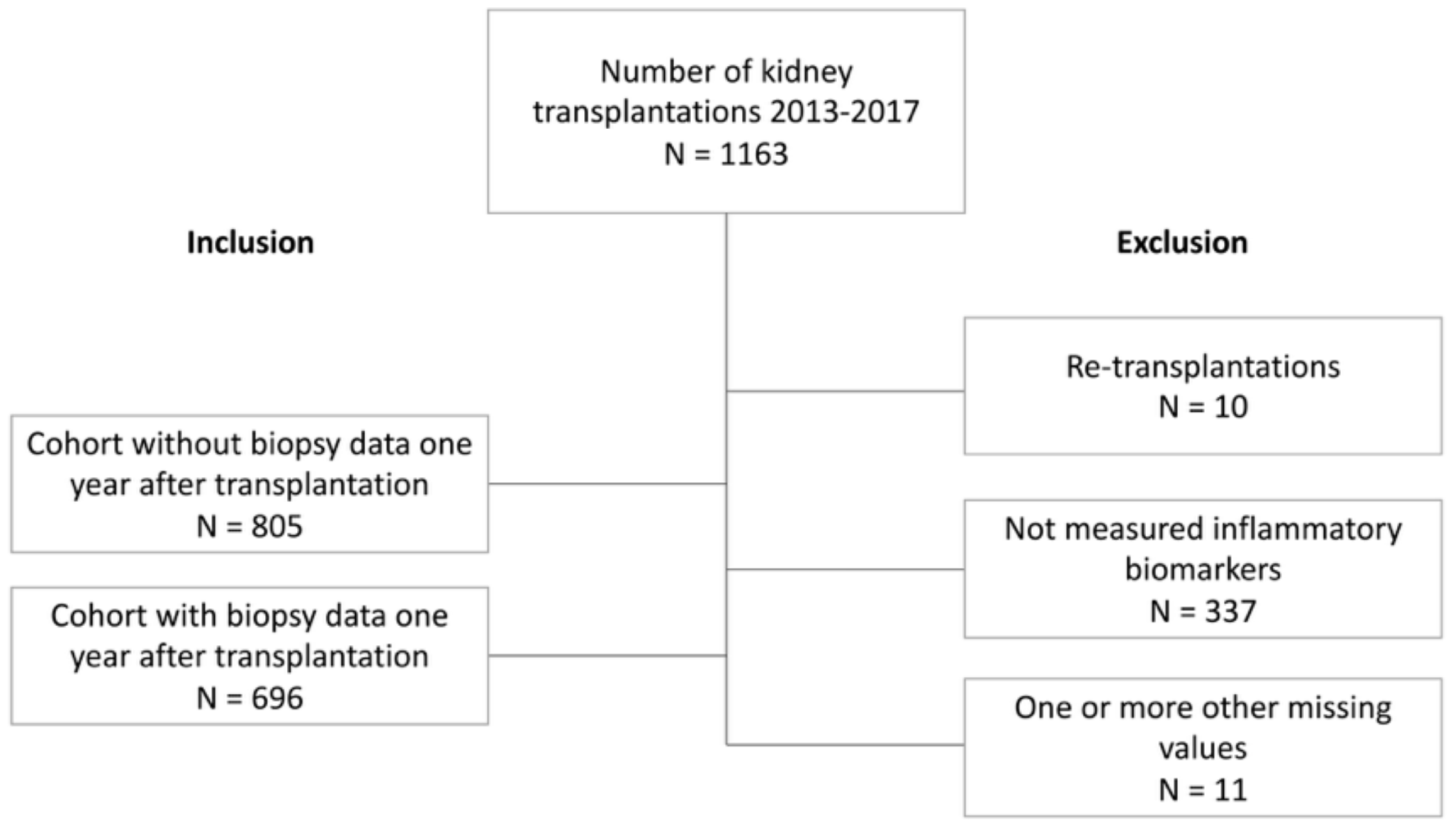
Flow chart.

This registry study included 805 patients who underwent kidney transplantation between 2013 and 2017 at Oslo University Hospital, Rikshospitalet, Norway. Only adult patients (> 18 years of age) with single kidney transplantation and patients who had been to the one-year follow-up were included (**Figure 1**). The baseline of the study was the one-year follow-up date. In all patients, a selected panel of inflammatory biomarkers was measured in blood samples taken from the patients at eight weeks- and one year after transplantation during planned surveillance follow-ups. At the time of measurement of the biomarkers the patients were clinically stable without ongoing systemic infections or rejection episodes. Of the included patients, 696 (86.5%) underwent a protocol kidney graft biopsy in relation to the one-year follow-up. Eleven patients (1.3%) were excluded because of one or more missing values for the variables included in the models. Patients were defined as immunological intermediate risk if they were panel-reactive antibody (PRA) positive and human leukocyte antigen (HLA) donor-specific antibody (DSA) negative. Patients with ABO-incompatible (ABOi) transplantation or DSA positive were classified as immunologically high risk. The study was conducted in line with the STROBE guidelines for observational studies and TRIPOD+AI (Transparent Reporting of a Multivariable For Individual Prognosis or Diagnosis + Artificial Intelligence) guidelines for studies on prediction models (16).

The primary outcomes were death-censored graft loss defined as the patients return to dialysis or re-transplantation, and death. Patients who died with a functioning graft were censored at the time of death as a functioning allograft. The causes of death are based on the local physician’s report on the death certificate. The ‘other causes’ category includes a range of conditions, as well as cases with ‘no obvious cause’ or ‘unknown cause of death’. The outcome data were retrieved from the National Renal Registry on July 18^th^, 2023.

### 2.2 Measurement of inflammation biomarkers and construction of the inflammation score

We measured five inflammatory biomarkers, which in our previous studies were associated with long-term graft loss and mortality (4, 5, 7): soluble tumor necrosis factor receptor 1 (sTNFR1), growth/differentiation factor 15 (GDF-15), CXCL16, osteopontin, and terminal 5b-9-complement complex (TCC). We collected plasma and serum samples at the follow‐up visit eight weeks and one year after kidney transplantation. The samples were stored at -80°C and thawed < three times. sTNFR1, GDF-15 CXCL16 and osteopontin levels were measured by enzyme immunoassays (EIA) using commercially available antibodies (R&D Systems, Minneapolis, MN) in a 384-well format using a combination of a SELMA (Jena, Germany) pipetting robot and a BioTek (Winooski, VT) dispenser/washer. Absorption was read at 450 nm with wavelength correction set to 540 nm using an EIA plate reader (Bio-Rad, Hercules, CA). TCC was measured by EIA using a monoclonal antibody aE11 reacting with a neoepitope exposed in C9 when incorporated in TCC (17, 18). Intra- and inter-assay coefficients of variation were < 10% for all assays, based on the performance in the laboratory that set up these analyses. In the final study population, no patients had values below the level of detection (5). CXCL16, sTNFR1, GDF-15 and TCC have shown associations with both graft loss and mortality, whereas osteopontin was only associated with graft loss. The biomarkers reflect several pathways including complement activation, TNF-activity, extracellular matrix remodeling, and vascular inflammation.

Inflammatory biomarker values were incorporated into the inflammation score. We used a quartile-based approached rather than clear cut-off values, as the measurement of the biomarkers is not standardized and is currently only meant for use in research. Thus, a value within the upper quartile of each biomarker was assigned one point, and accordingly, the inflammation score graded from 0 to 5. Biomarkers were equally weighted based on their individual risk coefficients (4, 5). Moreover, the values can only be strictly compared with samples analyzed at the same time as dilution can differ, but the distribution will remain the same. This implies that the values in this cohort cannot be directly compared to those from the 2007-2012 cohort.

### 2.3 Biopsies and histological classification

The goal for sufficient quality of the cores for histology was the presence of ten glomeruli and two or more arteries, but a minimum of seven glomeruli and one artery was accepted. Biopsies were graded according to the Banff 2019 guidelines (19). For application in the Cox regression models, the biopsy results were also graded as interstitial fibrosis and tubular atrophy (IFTA) (ci + ct), microcirculation inflammation (g and ptc), interstitial inflammation (i and t), and transplant glomerulopathy (cg), as in the iBox scoring system (10).

### 2.4 Candidate predictors

In the models on death-censored graft loss, we included the variables from the iBox score and added the inflammation score. We tested the models with and without histological data. The included variables were estimated glomerular filtration rate (eGFR) calculated using the 2021 CKD-EPI equation (20), urine protein-creatinine ratio (U-PCR), DSA-status, and the inflammation score. The histological data included were IFTA, microcirculation inflammation, interstitial inflammation, and transplant glomerulopathy (10).

In the model with mortality as the outcome variable, we included recipient age, smoking status, dialysis vintage prior to the first transplantation, either pretransplant diabetes mellitus or PTDM, and the inflammation score. These variables were chosen as they have previously been shown to be associated with long-term mortality (4). HbA1c and cholesterol levels, as well as the presence of DSA, were tested in the model but did not affect the results. The measurement of the inflammatory biomarkers eight weeks and one year after transplantation was paired, meaning that all included patients were alive and had a functioning graft one year after transplantation. Thus, we did not test the predictive abilities of the inflammation score at eight weeks after transplantation as the results would have been skewed.

In summary, the models were:

1. Model on graft loss with histological data: eGFR, logarithmic urinary protein creatinine ratio, DSA-status, inflammation score, IFTA, microcirculation inflammation, interstitial inflammation, and transplant glomerulopathy.
2. Model on graft loss without histological data: eGFR, logarithmic urinary protein creatinine ratio, DSA-status, and inflammation score.
3. Model on mortality: recipient age, smoking status, dialysis vintage, pretransplant diabetes or PTDM, and the inflammation score.

### 2.5 Statistical analyses

We used R version 4.3.2. All hypothesis tests were two-sided, and the significance level was set at 0.05. Continuous variables were described by means and standard deviations or by medians and interquartile ranges. We compared the means and proportions between patients with and inflammation score ≤ 1 and patients with an inflammation score > 1 by using Student´s t-test, analysis of variance, or the Chi-Square test. The difference in values of the inflammation biomarkers eight weeks and one year after transplantation was examined by paired Student T-test. Mortality and graft survival were estimated using the Kaplan-Meier method. In the Cox regression models, death-censored graft loss and mortality were outcome variables. When not specified, the hazard ratios reported were from the model including the continuous inflammation score. The proportional-hazards assumptions were tested by PH-tests. Nonlinear relationships between continuous variables and the outcome variables were investigated using restricted cubic splines (using the “rms” package in R) (21). In the model considering mortality there were no nonlinear relationships, but between graft loss and U-PCR nonlinearity was present. The U-PCR was transformed using the logarithmic variable. The included variables are described in the “Candidate predictors” section. The inflammation score was assessed as both a continuous- and a categorical variable. The median follow-up time was 6.5 years from the baseline assessment, and thus we tested the models´ calibration at six years.

We assessed the models´ discrimination ability by measuring the Harrell´s C-statistic by the “cindex” function in the “pec” package in R. We created Receiver Operating Characteristic (ROC) curves to illustrate the difference between the models with and without the inflammation scores by means of the “timeROC” package in R. The calibration of the model was evaluated by visual examination of calibration plots generated by using the “rms” package in where the predicted proportions were compared to the observed proportions estimated by the Kaplan-Meier method (22). Internal validation of the models was performed by using a bootstrap procedure where we resampled the original dataset created 1000 new datasets. In these datasets we simulated the performance analyses and calculated the optimism corrected c-statistic (22). We performed this for the final prediction models regarding death-censored graft loss with and without biopsy data, and mortality.

The difference between the models with and without added inflammation scores, and the fraction of new information added by implementing the inflammation score into the models were tested by performing likelihood ratio (LR) χ^2^ tests. An estimation of the added prognostic value was done by measuring the adequacy of the model (1- [Pre-test LRχ^2^/Post-test LRχ^2^]) (23, 24). Additionally, we compared the c-statistics of the models with and without the inflammation scores against each other using a bootstrapping procedure. Finally, we performed decision curve analyses to quantify and illustrate the additional clinical benefits of adding the inflammation score (“dcurves” package in R) (25). In the plots, the “Net Benefit” (the proportion of true positives in the absence of false positives) is on the y-axis and Threshold Probability on the x-axis. Test tradeoffs were calculated by 1/ΔNet Benefit at a given threshold.

## 3 Results

### 3.1 Study population: characteristics, outcomes, and biomarker distribution

Baseline characteristics are presented in **Table 1**. The main cohort with inflammatory biomarkers consisted of 805 patients, and of these, histological data were available in 696 patients. The median follow-up time was 6.4 (interquartile range 5.1-7.8) years. There were 168 (20.9%) deaths and 42 (5.2%) graft losses during the follow-up period in the main cohort, and 37 (5.3%) graft losses in the biopsy cohort. The causes of death were categorized as CVD (n=37, 22.0%), malignancies (n=46, 27.4%), infections (n=31, 18.5%), and other causes (n=54, 32.1%). The total number of deaths correlated with the inflammation grade with a similar distribution observed across all causes of death.

**Table 1:** Study population characteristics.

Inflammation score			
General characteristics	0-1 (n=588)	≥2 (n=217)	P-value
Recipient age, y (SD)	48.8 (16.2)	57.3 (14.9)	<0.001
Male sex, yes	394 (66.9%)	154 (71.0%)	0.31
Smoking history, yes	271 (46.0%)	116 (53.5%)	0.27
Pretransplant diabetes, yes	63 (10.7%)	49 (22.6%)	<0.001
PTDM, yes	46 (8.5%)	28 (10.5%)	0.25
Dialysis vintage, months (SD)	12.1 (10.2)	16.5 (18.4)	0.004
Cause of graft loss			
Rejection	9 (64.4%)	13 (46.4%)	
Recurrence of original disease	3 (21.4%)	7 (25.0%)	
Other	2 (14.3%)	8 (28.6%)	
Transplant characteristics			
Donor age, y (SD)	47.8 (16.4)	62.3 (12.7)	<0.001
Male donor (%)	303 (51.4%)	104 (47.9%)	0.42
Deceased donor (%)	383 (65.0%)	190 (87.6%)	<0.001
Cold ischemic time, h (SD)	10.0 (5.8)	12.5 (6.7)	<0.001
Delayed graft function (%)	41 (7.0%)	42 (19.4%)	<0.001
Mean number of HLA-mismatches (SD)	3.0 (1.5)	3.1 (1.5)	0.55
ABOi (%)	18 (3.1%)	9 (4.1%)	0.59
DSA ^A^			
Preformed (%)	27 (4.6%)	13 (6.0%)	0.53
dnDSA ^B^ (%)	14 (2.4%)	4 (1.8%)	0.46
*Immunological risk*			
High (%)	21 (3.6%)	5 (2.3%)	0.34
Intermediate (%)	20 (3.4%)	10 (4.6%)	
Tacrolimus C0 (ug/mL)	6.1 (1.8)	6.3 (1.9)	0.12

^A^DSA within the first year after transplantation. ^B^ *de-novo* DSA.

**Figure 2:**
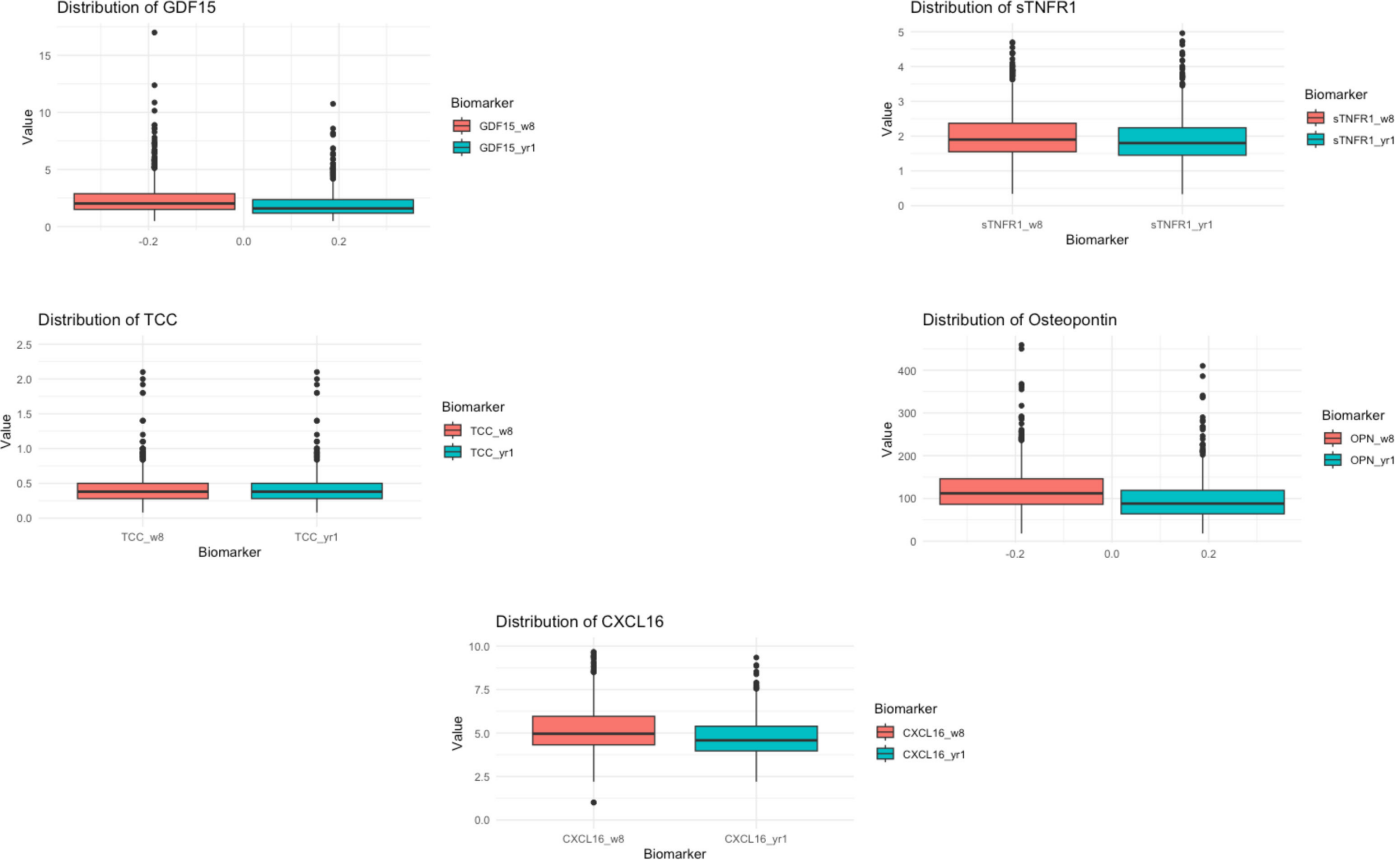
Distribution of the inflammatory biomarkers eight weeks and one year after kidney transplantation. Boxplots showing the distribution of the inflammatory biomarkers eight weeks and one year after kidney transplantation. The values after eight weeks are highlighted in red, and the one-year data is in blue. The scale of the y-axis is adjusted according to the variance of the appropriate biomarker.

We investigated the variance among biomarker values from eight weeks to one year after transplantation (**Figure 2**). Overall, the biomarker levels were significantly lower after one year compared with eight weeks after transplantation (P < 0.001): (sTNFR1: 1.90 [IQR 1.56-2.38] ng/ml and 1.80 [IQR 1.45-2.25] ng/ml, GDF15: 2.02 [IQR 1.50-2.88] ng/ml and 1.59 [IQR 1.17-2.36] ng/ml, CXCL16: 4.97 [IQR 4.33-6.02] ng/ml and 4.59 [IQR 3.97-5.40] ng/ml, osteopontin 112 [IQR 86.3-146] ng/ml and 88 [IQR 64-119] ng/ml and TCC: 0.38 [IQR 0.28-0.51] Complement Activating Units [CAU] and 0.37 [IQR 0.29-0.50] CAU; eight weeks and one year, respectively).

### 3.2 Sensitivity analysis, internal validation, and calibration of the models

#### 3.2.1 Death-censored graft loss with histological data

A total of 696 (86.5%) patients had a biopsy performed one year after transplantation. There were 39 (5.6%) subsequent graft losses in this group. We applied the variables identified in the iBox model as the baseline for our model and added the systemic inflammation score based on one-year biomarker samples (10). **Table 2** presents the results of the Cox regression models. Significant associations with graft loss were observed for the inflammation score both as continuous (HR 1.62, P = 0.001) and categorical (HR 6.61, P < 0.001) variables, as well as the presence of DSA (HR 4.07, P = 0.003) and IFTA grade 3 (HR 4.04, P = 0.002).

**Table 2:** Multivariable cox regression analyses: graft loss with biopsy data, graft loss without biopsy data, and mortality.

	Hazard Ratio	95% Confidence Interval	P-value
Graft loss with biopsy data			
Inflammation score:			
Continuous	1.62	1.25-2.11	0.001
Categorical (0-1 ref):			
2-3	2.32	0.98-5.54	0.06
4-5	6.61	2.46-17.74	< 0.001
eGFR (ml/min/1.73m^2^)	0.98	0.96-1.01	0.19
Logarithmic u-PCR (mg/mmol)	1.17	0.96-1.44	0.13
DSA^A^	4.07	1.58-10.5	0.003
IFTA^B^ grade 2	1.14	0.37.3.47	0.81
IFTA^B^ grade 3	4.04	1.67-9.70	0.002
Microcirculation inflammation (≥ 3)	1.12	0.09-13.3	0.92
Interstitial inflammation (≥ 3)	0.74	0.21-2.26	0.59
Transplant glomerulopathy (≥ 1)	2.99	0.73-12.32	0.13
Graft loss without biopsy data			
Inflammation score			
Continuous: Categorical (0-1 ref)	1.63	1.30-2.05	< 0.001
2-3	2.10	0.97-4.57	0.06
4-5	5.66	2.37-13.54	< 0.001
eGFR	0.98	0.96-0.99	0.03
Logarithmic u-PCR	1.27	1.09-1.48	0.003
DSA^A^	5.05	2.22-11.49	< 0.001
Mortality			
Inflammation score			
Continuous	1.38	1-25-1.53	< 0.001
Categorical (0-1 ref)			
2-3	2.10	1.49-2.96	< 0.001
4-5	3.14	2.02-4.88	< 0.001
Recipient age, y	1.05	1.04-1.07	< 0.001
Smoking history (yes)	1.31	1.08-1.58	0.005
PreDM or PTDM (yes)	1.67	1.23-2.27	0.001
Dialysis vintage, m	1.01	1.00-1.02	0.002
HbA1c ^C^	1.00	0.98-1.03	0.84
Total cholesterol ^C^	0.99	0.87-1.12	0.82
DSA ^A,C^	0.43	0.13-1.39	0.16

^A^Presence of DSA during the first year, either preformed or *de novo* DSA. ^B^Presence of IFTA changes in biopsies (grade 2 or grade 3). No IFTA or grade 1 was used as the reference category. ^C^Not included in the final model.

**Figure 3:**
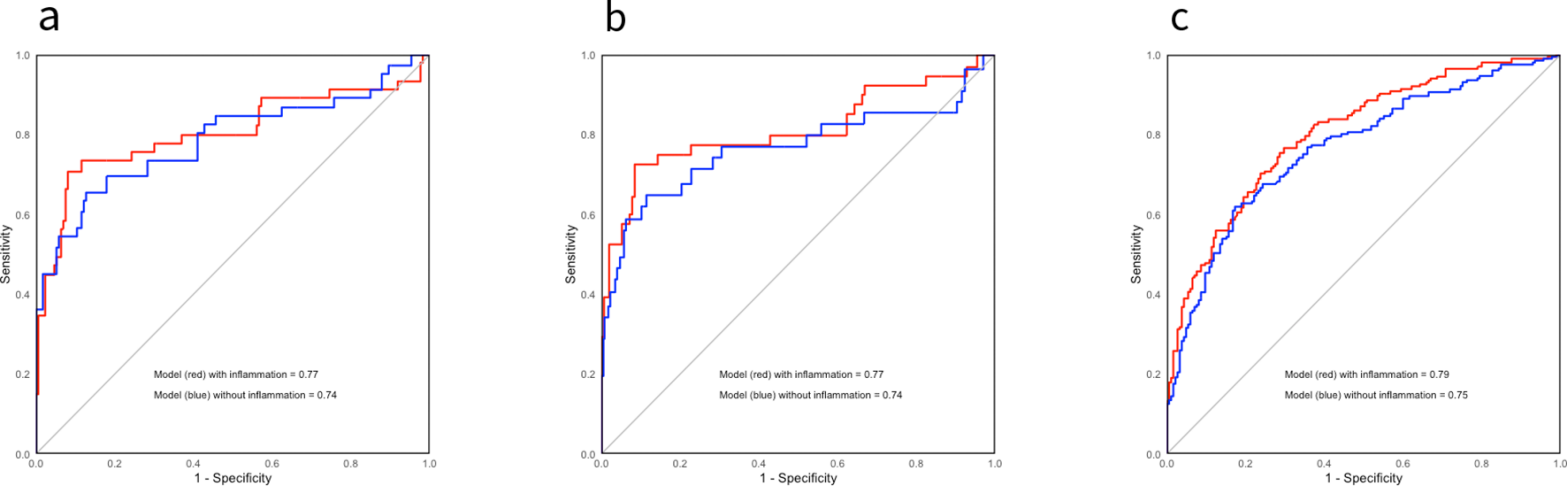
Sensitivity analyses: Receiver operator characteristics curves displaying the discrimination ability of the different models six years after inclusion. ROC-curves displaying the discrimination ability for the different models on kidney graft loss with histological data **(A)** and without histological data **(B)**, and mortality **(C)**. The models that include the inflammation score are highlighted in red, and the ones without the inflammation score are in blue. The c-statistics are displayed in the figure.

**Figure 4:**
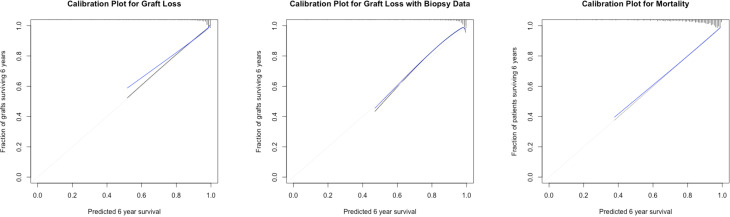
Calibration plots six years after kidney transplantation. Calibration plots at six years for kidney graft loss and mortality. The y-axis displays the observed proportions of kidney grafts or patients six years after transplantation (estimated by the Kaplan-Meier method), whereas the x-axis shows the predicted probabilities. The black line illustrates a perfectly calibrated model, and the blue line represents the optimism corrected appropriate model.

We performed sensitivity analyses of the model and calculated a c-statistic value of 0.77. We additionally created ROC-curves at six years after inclusion (**Figure 3**). The internal validity of the model was tested by a bootstrapping procedure (B=1000). The optimism corrected c-statistic was 0.76. We tested the model´s calibration at six years by visual examination of calibration plots (**Figure 4**). For this model there was some overfitting, but the overall calibration was acceptable. The c-statistic of the model without the inflammation score (the model representing the iBox score) was 0.74. Likelihood ratio tests indicated that the addition of the inflammation score in the model significantly enhanced the model fit with up to 20% of new prognostic information (**Table 3**). A direct comparison of the c-statistics for the model with and without the inflammation score did not show a significant difference (P = 0.17).

**Table 3:** Fractions of new information added from including the inflammation score in the different models ^A^.

	Fraction of new information added (%)		*P value (likelihood ratio test)*	
	Graft loss	Mortality	Graft loss	Mortality
Final model, linear ^B^	23.0 %	26.9 %	< 0.001	< 0.001
Final model, categorical ^C^	24.6 %	22.3 %	< 0.001	< 0.001
Model with histological data, linear	20.5 %		< 0.001	
Low risk model without histological data, linear ^D^	31.3 %		< 0.001	

^A^The proportion of the total predictive information added by including the inflammation score into the models by measuring the pre- and post- likelihood ratio tests.

^B^Final model on graft loss and mortality including the inflammation score as a linear variable.

^C^The final model on graft loss and mortality including the inflammation score a categorical variable.

^D^Excluding patients with intermediate- and high immunological risk.

#### 3.2.2 Death-censored graft loss without histological data

The primary cohort consisted of 805 patients. The model used without histological data consisted of four variables that were all significantly associated with graft loss: the continuous inflammation score (HR 1.63, P < 0.001)/categorical inflammation score (HR 2.3-6.6, P < 0.001), eGFR (HR 0.98, P= 0.03), the logarithmic value of u-PCR (HR 1.27, P = 0.003), and the presence of DSA (HR 5.05, P < 0.001). We tested the model´s discrimination ability, and the c-statistic value was 0.77. We verified the internal validity again using a bootstrapping procedure, and the optimism corrected C-statistic was 0.75. The calibration six years after transplantation was acceptable (**Figure 4**). When we excluded the inflammation score from the model the C-statistic was 0.74, but there was no significant change by direct comparison of the C-statistics (P=0.12). However, the added prognostic information when also including the inflammation score was 23% (continuous) and 24% (categorical) and was significantly different (P < 0.001) by comparing likelihood ratios (**Table 3**).

We also tested the model in patients who were defined as low-risk (PRA-negative, DSA-negative, and ABO-compatible transplantations) at the time of transplantation (n=651, graft losses = 33). In this population, there was no difference in the predictive abilities between the score with histological data (C-statistic=0.79) and the score without histological data (C-statistic=0.79). The optimism-corrected C-statistic was 0.78 with histological data and 0.77 without histology. Implementation of the inflammation score significantly improved the model fit (**Table 3**).

#### 3.2.3 Mortality

A total of 168 (20.9%) deaths occurred during the follow-up period. In the model describing long-term mortality, we included risk factors identified in our previous studies (4): recipient age, smoking status, dialysis vintage, pre-transplant diabetes or PTDM, and the systemic inflammation score. All variables were retested in Cox regression models and were found to be significantly associated with long-term mortality (**Table 2**). Total cholesterol levels, HbA1c, and the presence of DSA were also tested in the model, but they did not affect the results.

The C-statistic of the model was 0.79. These results were validated through the same procedure as previously described, and the optimism-corrected C-statistic was 0.77. The calibration of the model six years after transplantation was adequate, supporting the validity of the model. The c-statistic of the model without the inflammation score was 0.74. Likelihood ratio tests indicated that the model significantly increased the fit of the model (P < 0.001) and contributed up to 22-27% of new prognostic information by comparing likelihood ratios (**Table 3**, **Figure 3**). A direct comparison of the C-statistics also showed a significant difference (P = 0.001).

### 3.3 Decision curve analyses

**Figure 5:**
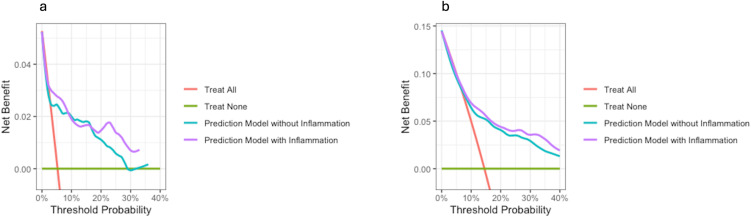
Decision curve analyses. Decision curves on graft loss **(A)** and mortality **(B)**. The net benefit is on the y-axis and the threshold probability on the x-axis. The prediction model including the inflammation score is in purple. The red line represents a scenario where all patients are treated, and the green line illustrates a scenario where none is treated. In all scenarios, both models with and without the inflammation score perform better than the “treat all” approach. For both graft loss and mortality the model with the included inflammation score performs better.

**Figure 5** shows the decision curves that illustrate the added benefit achieved by implementing the inflammation score into the models. Regarding graft loss (a) there is no apparent difference up to a risk threshold of approximately 20%, whereas at higher risk thresholds, there is a benefit of adding the inflammation score to established risk factors. For mortality (b), there is a slight benefit associated with adding the inflammation score to the model from the 10% threshold and upwards. At a 35% threshold regarding mortality, the test trade-off [minimum number of tests required to achieve one additional true positive (25)] was 71 (1/0.014) and 140 (1/0.014) at 10%. Regarding graft loss, at the 30% threshold the test trade-off was 77 (1/0.013).

## 4 Discussion

In this study, we have demonstrated the association between systemic inflammation one year after transplantation and both long-term death-censored graft loss and mortality in an updated cohort recruited between 2013 and 2017. This complements findings from cohorts suggesting the importance of inflammation early after transplantation and adverse outcomes (4, 5). Implementation of systemic inflammation, assessed by a predefined inflammation score representing several distinct pathways, into predictive models added to the prognostic discrimination abilities regarding both mortality and death-censored graft loss, thus suggesting a role for non-invasive systemic inflammation scores in the risk assessment of kidney transplant recipients after transplantation. These results are supported by findings in other populations that represent either direct or indirect measurements of systemic inflammation on adverse outcomes following kidney transplantation (6, 7, 26, 27).

The biomarkers included in the final inflammation score are associated with either kidney graft loss or mortality following kidney transplantation (4, 5, 7). They represent activation of TNF related pathways (sTNFR1), complement activation (TCC), chemotaxis (CXCL16), vascular inflammation (CXCL16 and sTNFR1), and extracellular matrix remodeling (GDF-15 and osteopontin). TCC, sTNFR1, and CXCL16 are additionally markers of innate immunity. Lamarthée et al. demonstrated the presence of innate immune cells in biopsies with AMR supporting the role of innate immune cell involvement and allorecognition in rejection (14, 15), and innate immune activity is associated with poor outcomes in kidney transplant recipients with infections (28).

By combining biomarkers reflecting several pathways into one score, we minimized the risk of disregarding or not identifying the relevant pathways. The iBox Scoring System was used as the basis of the prediction model for kidney graft loss. The performance of the model was very good both with (C-statistic=0.77) and without histological data (C-statistic = 0.77), and the performance of the model improved significantly after the implementation of the inflammation score compared to the base model. The discrimination abilities were slightly inferior compared to those in the iBox validation cohorts (10), which can probably be explained by the rather low number of events (n=42).

One of the main strengths of the inflammation score is its association with both graft loss and mortality. There are many models predicting the risk of graft loss, yet there is no adequate model for patient survival implemented in daily practice. Our model for long-term mortality included the inflammation score in addition to the established risk variables recipient age, smoking history, pre- or post-transplant diabetes, and dialysis vintage (4). The model performed adequately (C-statistic=0.79) in our population, and the model performed significantly better after the inflammation score was included. This is also illustrated in the decision curves (**Figure 5**). In contrast, cholesterol and HbA1c levels did not come out significantly in the Cox regression analyses and did not add to the discrimination ability. We also tested other transplant specific risk factors in the model (DSA), but these did not affect the results. DSA is currently the most important single biomarker related to rejection and death-censored graft loss, however, it is not associated with mortality. Donor-derived cell-free DNA (dd-cf DNA) is an emerging biomarker following transplantation. A recent study found associations between circulating dd-cf DNA and the development and severity of both TCMR and AMR (29), but there is currently not described any associations between dd-cf DNA and mortality.

The role of protocol biopsies in the follow-up after kidney transplantation is disputed. A recent study from Norway and Finland found limited utility of one-year protocol biopsies in low-risk patients without any previous events (e.g. acute rejection, DSA) (30). In our study, there were no apparent differences between the predictive abilities of the models also with and without histological data. In addition, when we only assessed patients having a low immunological risk at the time of transplantation, there was no difference between the two approaches. We previously described a correlation between systemic vascular inflammation and local inflammatory changes in the kidney graft and between a score representing extracellular matrix regulation and IFTA changes in the graft (5). In “low-risk” patients, the measurement of the systemic inflammation score could be a better and non-invasive tool to identify patients at risk of graft loss than protocol biopsies.

Based on the study design we cannot establish whether the inflammation represents a marker of disease activity, is the driving force of the disease, or a combination thereof. It is also possible that an increased inflammatory score is a result of ineffective immunosuppression or wrongly targeted immunosuppression, and if so, could be a signal for adjusting the actual immunosuppressive regimen in specific patients. Structured data on infections and hospital admissions were not available and thus we could not test the associations between the inflammation score and the risk of future infections. The score has not undergone proper external validation, but the associations between the inflammation score and both mortality and graft loss have been reproduced in two different cohorts and datasets from different time eras at our center. Outside of TCC, the measurement of the biomarkers is currently not standardized and can only be compared to the values measured at the same time. However, the levels of these markers were generally relatively high (> 10 ng/ml) and the way to routine measurements should not be too long. Ideally, we would have wanted more events regarding graft loss. The low number of events in the graft loss model is a limitation, although the findings are highly significant.

In conclusion, a pre-defined systemic inflammation score representing several molecular pathways is associated with both long-term graft loss and mortality in kidney transplant recipients. When implemented into prediction scores with clinical data, the performance of all models was significantly enhanced after implementation of the inflammation score. The model on mortality after kidney transplantation performed better than any model known to us. Our results suggest that structured evaluation of systemic inflammation, and potentially repeated measurements within the first year after transplantation, could be an important tool for risk assessment of kidney transplant recipients and help identify risk patients at an early stage.

## Data Availability

De-identified data may be shared upon reasonable request and after application to the Regional Committee for Medical and Health Research Ethics (REK Sørøst, Norway), in cooperation with the authors. Requests to access the datasets should be directed to TH, email: torbjorn.heldal@hotmail.com.
